# Length of time periods in treatment effect descriptions and willingness to initiate preventive therapy: a randomised survey experiment

**DOI:** 10.1186/s12911-018-0662-2

**Published:** 2018-11-20

**Authors:** Erik Berglund, Ragnar Westerling, Johan Sundström, Per Lytsy

**Affiliations:** 1 Department of Public Health and Caring Sciences, Uppsala University, Box 564, SE-751 22 UPPSALA, Sweden; 2 Department of Medical Sciences, Uppsala University, Uppsala, Sweden

**Keywords:** Medical decision-making, Risk communication, Risk perception, Necessity-concern framework

## Abstract

**Background:**

Common measures used to describe preventive treatment effects today are proportional, i.e. they compare the proportions of events in relative or absolute terms, however they are not easily interpreted from the patient’s perspective and different magnitudes do not seem to clearly discriminate between levels of effect presented to people.

**Methods:**

In this randomised cross-sectional survey experiment, performed in a Swedish population-based sample (*n* = 1041, response rate 58.6%), the respondents, aged between 40 and 75 years were given information on a hypothetical preventive cardiovascular treatment. Respondents were randomised into groups in which the treatment was described as having the effect of delaying a heart attack for different periods of time (Delay of Event, DoE): 1 month, 6 months or 18 months. Respondents were thereafter asked about their willingness to initiate such therapy, as well as questions about how they valued the proposed therapy.

**Results:**

Longer DoE:s were associated with comparatively greater willingness to initiate treatment. The proportions accepting treatment were 81, 71 and 46% when postponement was 18 months, 6 months and 1 month respectively. In adjusted binary logistic regression models the odds ratio for being willing to take therapy was 4.45 (95% CI 2.72–7.30) for a DoE of 6 months, and 6.08 (95% CI 3.61–10.23) for a DoE of 18 months compared with a DoE of 1 month. Greater belief in the necessity of medical treatment increased the odds of being willing to initiate therapy.

**Conclusions:**

Lay people’s willingness to initiate preventive therapy was sensitive to the magnitude of the effect presented as DoE. The results indicate that DoE is a comprehensible effect measure, of potential value in shared clinical decision-making.

## Background

Insufficient adherence to cardiovascular treatments is considered an important factor in treatment failure [1], and insufficient adherence may increase cardiovascular morbidity [2, 3]. Several studies link patient/person-centred care (PCC) to adherence, as part of a successful preventive medical treatment strategy [4, 5]. To enable optimal adherence, using a patient-centred approach and informed decision-making, patients need to receive treatment information that is both correct and easy to understand. Statistical effect measures from clinical trials are known to influence patients’ views and understandings of a treatment, and it has been shown that the format of the effect measure communicated influences decisions by both physicians and patients [6–9]. The most common measures used to describe a preventive treatment effect include relative risk reductions (RRR) and absolute measures such as absolute risk reductions (ARR) and numbers-needed-to-treat (NNT) [10]. These measures are proportional, i.e. they compare the proportions of events in relative or absolute terms. RRRs have, compared to ARRs and NNTs been shown to score more favourably in evaluations of treatments [10, 11]. RRRs, however, does not account for of the baseline risk and are, thus, alone not sufficient to distinguish a treatment effect that is clinically valuable from one that is not. Furthermore, different magnitudes of RRRs do not seem to clearly discriminate between levels of effect presented to lay people [12]. It has been argued that the amplification effect, following a RRR effect description, biases or misleads patients towards accepting interventions in a way that is not consistent with their own values [13, 14]. For that reason, RRRs are not alone recommended in shared decision making. In a search for effect measures that are appropriate for communication of risk and treatment effect in the preventive setting, different measures depicting postponement of events, time-to-event or prolongation of life have been suggested [15–18]. A gain in disease free time or a gain in life expectancy estimates treatment effect in a way that might be of intuitive value to patients. Lay people seems to have the capacity to determine and choose between different levels of treatment effect in settings such as hip fracture [17] and heart attack [18], when the treatment effect is explained as postponements of time to events. Such time estimations studies have, hitherto, used hypothesised or extrapolated data, which proves the concept of time-based measures, but provides little value for risk and effect communication in clinical practice. A set of complementary measures that go beyond the proportional measures has recently been proposed, that involve assessing between-group differences in percentiles of the survival function [19–21]. By the use of Laplace regression modelling [22], it is possible to assess a treatment effect as the time an event is delayed due to treatment, which can be expressed as the Delay of Event (DoE) measure [19]. The DoE measure evaluates the time difference between two treatment arms at equal event rates. Over the follow-up period, it presents a curve of how the treatment effect develops over time, which has been exemplified using data from clinical studies [19, 21]. The DoE is an absolute measure and conditional on the event. This means that for a study participant who would have an event during the follow-up without treatment, the DoE depicts the time of the delay when treated. A person who does *not* experience the event of interest during a follow-up cannot benefit from treatment during this period. The DoE is a complimentary effect measure, and recent results suggest that the DoE compares well to RRR and better than ARR when measuring laymen’s willingness to initiate a hypothetical preventive treatment [20].

The objective of this study was to investigate if the length of time in the DoE measure influences lay people’s willingness to initiate a preventive treatment against cardiovascular disease (CVD). Secondary objectives were to investigate how the different magnitudes of DoE treatment effects affect individuals’ understanding and confidence in the medication and their willingness to pay for treatment (WTP). We hypothesised that people’s preference for a preventive therapy is positively influenced by the length of the delayed disease when it is expressed in terms of DoE.

## Methods

### Design and sample

This is a cross-sectional randomised survey experiment [23, 24], in a population-based sample, aged 45–75 years. Eighteen hundred (1800) randomly selected persons from the Swedish national population registry were randomly allocated into three equally sized groups. The groups received information about a hypothetical treatment’s effect as three different delayed event times of heart attack: 1 month, 6 months or 18 months and were then asked different follow-up questions. The questionnaire was returned by 1041 individuals; 23 persons could not be reached or declined to participate, and 736 did not answer, making the response rate of the distributed questionnaires 58.6% (1041/1777). The response rate in the three groups varied from 56.8 to 59.8%. Data were collected from November 2013 to February 2014. A previous study compared the 18 months DoE arm to proportional effect measures [20].

### Background questions

Background data were assessed using questions about the respondent’s gender (dichotomous variable), age (continuous variable), and educational level (categorised in three groups).

### History of cardiovascular disease

Data were collected using questions about history of CVD (dichotomised to having had myocardial infarction and/or angina, or not); these questions have been used in previous studies [25, 26].

### Beliefs about medicines

Patients beliefs was assessed using the Necessity-Concern Framework (NCF) which was developed specifically to address views of drug treatments [27]. According to the NCF, a patient’s decision regarding approving and adhering to a treatment is the result of a trade-off between the patient’s perceived need for a prescribed treatment (necessity) and their worries about potential adverse effects as a result of the treatment (concern). NCF has been used in a broad range of different quantitative studies exploring drug treatment adherence [28–31], including cardiovascular diseases [32–35]. NCF was assessed by using the Beliefs about Medicines Questionnaire (BMQ) in specific version [27]. The BMQ questionnaire has been translated into Swedish and has been used previously in Sweden [26, 36–39].

### Hypothetic scenario and treatment effect information

The study was conducted as an information intervention with randomised groups in a survey experiment. Respondents were asked to imagine a scenario where they had an increased risk of CVD, and that their physician suggested a preventive cardiovascular drug treatment (comparable to statin treatments). The text was outlined: “Imagine that in the next five years you will have an increased risk of having a heart attack. Your physician offers you a treatment with rare and mild side effects, to be taken in pill form once a day. The usefulness of the treatment has been evaluated in scientific studies, and the effect can be described as follows: If you would have a heart attack in the next 5 years it will be delayed for up to [1 month] if you take the treatment.” The text describing the effect measure, in square brackets above, expressed different magnitudes of the Delay of Event measure. The first group received a treatment effect described as 1 month DoE, the second group 6 months DoE, and the third group 18 months DoE.

### Outcome variables

Information about willingness to initiate treatment, the dependent variable in the analyses, was assessed using the question: “If you were in the same situation as the person in this case would you take the treatment?”. Response alternatives were yes or no. Other questions that were used to evaluate the views of the treatment were: “How much benefit do you assess that the drug would have?”; “Would you feel safe to take the drug?”; “Would you, based on the description, be motivated to take the medication on a daily basis?”; “How important is it to adhere to the treatment prescription?” and “To what extent does the description help you make a medical decision?”. The answers to these questions were assessed on seven-point Likert scales ranging from: 1 (not at all) to 7 (very much). Little is known about the monetary value that individuals assign in relation to the proposed medication. Willingness to pay (WTP) is an assessment of the highest price an individual will pay for a good or service [40]. WTP was assessed with an open-ended question: “What is the maximum amount of money that you would pay per month during a five-year period to receive the treatment?”. WTP was measured in Swedish currency (SEK), and for the purpose of this study was converted to Euros (€) at an exchange rate of 0.10.

### Statistical analysis

Chi-square analyses and multivariate logistic regression analyses were used to investigate associations between willingness to initiate therapy and the presented DoE magnitudes. Associations between lay people’s views of the treatment descriptions, WTP and presented DoE magnitudes were tested with Kruskal-Wallis H tests. Consent to initiate preventive therapy was tested in multivariate binary logistic regression models. A stepwise approach was performed to build these models. Model 1 included length of time delay and demographics. Model 2 included model 1, history of cardiovascular diseases and NCF. In order to examine the interaction effects of DoE and age group (categorised as over/under 60 years of age), multiplicative interaction terms of length of time delay × age group were included in model 3. Statistical Package for the Social Sciences (SPSS)® version 22 (IBM SPSS Statistics for Windows, Armonk, NY: IBM Corp Chicago, IL, U.S.A.) was used for descriptive statistics and statistical tests. A *p*-value ≤0.05 was considered statistically significant.

## Results

The study population was on average 61.8 years old and consisted of slightly more women than men. College or university was the most common completed education level. A distribution of demographic variables, overall and according to group allocation, is shown in Table 1. In the study population the prevalence of a history of cardiovascular disease (heart attack and/or angina) was 5.8%. Overall the three groups were well balanced in baseline characteristics.

**Table 1:** Distribution of characteristics among participants

		Delay of Events			
Category	Subcategory	1 month	6 months	18 months	Overall
Gender	Men, % (n)	48.6 (169)	40.2 (141)	44.3 (148)	44.3 (458)
	Women, % (n)	51.4 (179)	59.8 (210)	55.7 (186)	55.7 (575)
Age	Mean (SD)	61.7 (8.5)	61.8 (8.7)	61.8 (8.6)	61.8 (8.6)
Education level	Compulsory school, % (n)	27.2 (94)	29.0 (101)	27.5 (92)	27.9 (287)
	Secondary school, % (n)	35.9 (124)	35.3 (123)	35.8 (120)	35.7 (367)
	College or university, % (n)	36.8 (127)	35.6 (124)	36.7 (123)	36.4 (374)
History of cardiovascular disease	Have had heart attack or angina, % (n)	7.4 (25)	5.5 (19)	4.5 (15)	5.8 (59)
	No history of heart attack or angina, % (n)	92.6 (315)	94.5 (327)	95.5 (315)	94.2 (957)
Necessity-concern Framework	Necessity, median^a^ (mean)	12 (12.1)	11 (11.0)	12 (12.6)	12 (11.6)
	Concern, median^a^ (mean)	4 (4.8)	3 (4.4)	4 (5.1)	4 (4.8)

Figures as percentages and numbers (n) if not stated otherwise. Mean values are presented with standard deviation (SD)

^a^Necessity and concern was used in index form, ranging from 0 to 20

### Decision to initiate treatment and views of treatment

Overall, 65.4% of the respondents were willing to take the suggested medication. The proportions accepting the treatment were 80.5, 70.6 and 45.5%, for a DoE of 18 months, 6 months and 1 month, respectively. The difference was statistically significant between the three groups (Table 2).

**Table 2:** Treatment decision and evaluation of descriptions

	Delay of Events					
Outcome	1 month	6 months	18 months	Overall	CHI-Square	*P*-value
Willingness to initiate treatment, % (n)	45.5 (156)	70.6 (243)	80.5 (268)	65.4 (667)	98.71^a^	< 0.001
Benefit from medication, md (q1, q3), m	3 (2,5), 3.18	4 (2,5), 3.99	5 (4,6), 4.58	4 (2,5), 3.91	96.20^b^	< 0.001
Feel safe with medication, md (q1, q3), m	3 (2,5), 3.45	4 (2,5), 3.95	4 (3,6), 4.25	4 (2,5), 3.88	30.35^b^	< 0.001
Be motivated to take medication, md (q1, q3), m	2 (1,5), 3.17	4 (2,6), 4.09	5 (3,6), 4.66	4 (2,6), 3.97	79.64^b^	< 0.001
Importance to adhere, md, (q1, q3), m	5 (2,7), 4.47	6 (4,7), 5.27	5 (5,7), 5.67	6 (4,7), 5.13	41.91^b^	< 0.001
Willingness to pay (Euro), md (q1, q3), m^c^	5 (0, 16), 13.4	10 (5, 30), 23.2	20 (10,30), 25.7	10 (0, 30), 20.8	65.10^b^	< 0.001
Use of information, md (q1, q3), m	5 (3,6), 4.63	5 (4,6), 4.65	5 (4,6), 4.91	5 (4,6), 4.72	3.25^b^	0.20

^a^Pearson Chi-Square

^b^Kruskal-Wallis H test

^c^Willingness to pay was measured in Swedish krona (SEK) 1SEK/0.10 Euro (€)

There were statistically significant differences between the three groups due to the presented DoE regarding respondents’ views of the benefit from treatment, feeling safe to take the drug, motivation to take treatment, views on importance of adhering to prescription, and WTP. The longer time periods presented as DoE, the more favourable did the respondents consider the drug. The median WTP for the three different treatment descriptions ranged from €13.4 for DoE of 1 month, €23.2 for DoE of 6 months, to €25.7 for DoE of 18 months (*P* < 0.001). There was no difference in use of information from prescriptions (Table 2).

### Logistic regressions

The willingness to initiate therapy was further explored in logistic regression models (Table 3). The odds of being willing to initiate treatment increased with a longer DoE, in adjusted models: odds ratio (OR) 4.45 (95% confidence interval (CI) 2.72–7.30) for a DoE of 6 months, OR 6.08 (95% CI 3.61–10.23) for a DoE of 18 month compared with a DoE of 1 month (reference category). The results were similar also when interaction terms were included in the model: OR 5.51 (95% CI 2.47–12.30) for a DoE of 6 months and OR 5.69 (95% CI 2.44–13.27) for a DoE of 18 months. A higher perceived necessity of medication in medical treatments in the NCF slightly increased the odds (OR 1.07, CI 1.03–1.11) for being willing to initiate therapy. Having a history of cardiovascular disease (heart attack and/or angina) was significantly associated with having a higher willingness to initiate treatment in the crude and adjusted models. No statistically significant interaction effect was found between length of time delay and age group.

**Table 3:** Multivariate models of the odds of consenting to therapy

		Crude OR 95% CI	Model 1 OR 95% CI	Model 2 OR 95% CI	Model 3 OR 95% CI
Delay of Events (DoE)	Length of time delay				
	1 month (ref. cat.)	1	1	1	1
	6 month	2.88** (2.11 to 3.95)	3.01** (2.17 to 4.16)	4.45** (2.72 to 7.30)	5.51** (2.47 to 12.30)
	18 month	4.94** (3.50 to 6.97)	5.15** (3.62 to 7.33)	6.08** (3.61 to 10.23)	5.69** (2.44 to 13.27)
Demographic	Gender				
	Male (ref. cat.)	1	1	1	1
	Female	1.11 (0.85 to 1.43)	1.02 (0.77 to 1.35)	0.87 (0.56 to 1.33)	0.87 (0.57 to 1.32)
	Age group				
	under 60 years of age (ref. cat.)	1	1	1	1
	60 years of age or older	1.80** (1.35 to 1.28)	1.64** (1.12 to 2.20)	1.34 (0.87 to 2.07)	1.45 (0.76 to 2.70)
	Education level				
	University (ref. cat.)	1	1	1	1
	Secondary school or equal	1.43* (1.06 to 1.93)	1.54** (1.12 to 2.12)	1.13 (0.70 to 1.83)	1.12 (0.69 to 1.81)
	Compulsory school	2.28** (1.62 to 3.20)	2.19** (1.51 to 3.17)	1.87* (1.11 to 3.16)	1.89* (1.12 to 3.19)
History of cardiovascular diseases	History of heart attack or angina				
	No (ref. cat.)	1		1	1
	Had heart attack and/or angina	3.47** (1.62 to 7.41)		2.89* (1.13 to 7.41)	2.86* (1.11 to 7.36)
Necessity concerns framework^a^	Beliefs about medicine				
	Necessity	1.06** (1.03 to 1.10)		1.07** (1.03 to 1.11)	1.07** (1.03 to 1.11)
	Concern	1.03 (0.99 to 1.07)		1.00 (0.95 to 1.05)	1.00 (0.95 to 1.05)
Interaction effect^b^	DoE 6 month × 60 years of age or older	0.98 (0.52 to 1.87)			0.71 (0.26 to 1.92)
	DoE 18 month × 60 years of age or older	1.18 (0.58 to 2.39)			1.11 (0.38 to 3.22)

Odds ratio (OR), significance level and confidence interval (CI) for willing to initiate therapy (0 = “no”, 1 = “yes”)

^a^Necessity and concern was used in index form, ranging from 0 to 20

^b^Interaction effects was adjusted for variables included in the interaction term

* *P* ≤ 0.05, ** *P* ≤ 0.01 Model 1 = Length of time delay+demographics, Model 2 = Model 1 + History of cardiovascular diseases+Necessity concerns framework, Model 3 = Model 2+ interaction effect

## Discussion

This study had the objective of investigating whether the length of time in the Delay of Event measure affected lay peoples’ willingness to accept a proposed treatment. This study also aimed to investigate how the different time periods of delay affect individuals’ evaluation and confidence in a proposed treatment as well as their willingness to pay for a therapy with a certain stated effect.

A higher proportion of individuals were willing to accept the treatment when the effect was presented with a greater time period in DoE. These findings are in agreement with similar studies that have demonstrated that lay people’s choices are sensitive to length of time periods of a treatment effect when it is expressed as postponements in time [17, 18]. Time-based measures, such as DoE, are considered to be easier to comprehend than proportional measures [18]. The results in the present study suggest that time-based measures of treatment effects enable lay people to evaluate and choose between different magnitudes of delay, in contrast to RRR measures which do not help lay people to a sufficient extent [12].

Relative measures of risk reductions depict a perspective that is relevant to individual patients. Relative risk reductions are usually relatively stable over the follow-up time and across different risk groups, but they are not easily interpreted from the patient’s perspective. This may be because RRRs do not consider the time perspective, which is of profound meaning for patients. RRRs do not apply to the patients’ long-term perspective for events that eventually will occur, such as death or other major adverse events of which absolute risk increases with time. This is linked to the fact that RRRs does not account for the underlying absolute risk at baseline. On the other hand, absolute numbers, such as risk differences, are easy to grasp and emphasise the fraction of treated patients that do experience a benefit. Such an interpretation - that only some benefit - is, however, likely to be an underestimation of the effect, as ARRs and NNTs only consider treatment benefit that will persist over the time point of evaluation [41]. Absolute effect measures are further sensitive to the time period of evaluation as well as to the baseline risk of the study population, and is thus of unclear relevance for patients who are likely to be taking the medication on a different timescale than the study population. Time-based non-parametric measures such as the DoE can be favourably expressed as curves, which depict the development of the effect over time and may support well-informed treatment decisions.

The DoE measure is not an average that applies to everyone which is a potentially difficult aspect in a clinical situation. Rather it is an estimation of the time an event is delayed due to treatment for people who would have developed the event, if untreated. To assume that one will have the event might be a cognitive challenge, and there is a risk such an assumption will tend to inflate patients´ perceived necessity and concerns of the treatment. If so, it might bias treatment decisions in favour of taking the treatment. On the other hand, if a DoE effect is perceived as short, it might do the opposite. Clinical use of the DoE measure will provide patients and physicians an opportunity to reflect on several important aspects, such as the patient’s medical status, risk and the benefits from treatment, which is one of the suggested routines to achieve shared decision making within patient-centred care [42, 43]. It cannot be taken for granted that different populations will assess the DoE measure in equal ways. The absolute risk of having an event, as well as the perceived severity and consequences of having that event, might vary for different conditions over different populations. For instance, the risk of having an event following a chronic disease usually increases with age. At the same time older age might go with declining quality of life, which may affect the perceived importance of delaying (or avoiding) events. Hence, there is a need to investigate the appropriateness of present treatment effect as DoE, for different conditions and populations.

Our observations suggest that the length of the DoE affects several aspects of lay people’s view of the proposed treatment, including their degree of motivation for taking it. The magnitude of the DoE showed no influence on the perceived usefulness of the information provided; consequently the quality of information and the ability to be informed was not dependent on the presented effect. This indicates that the data and their presentation were useful for decision and evaluation of effect, regardless of the presented DoE magnitude. Respondents’ willingness to pay for the treatment was higher for longer time periods of DoE. However, the relationship seems to level off in a way sometimes referred to as “the law of diminishing marginal utility”, which means that the first unit of consumption of a goods or service yields more utility than the second and subsequent units. In this case lay people tended to have higher WTP for a month in a shorter total time period than for a month in a longer time period. Previous research has discussed diminishing marginal utility in relation to gain of future life-years compared to those at present age. People tend to value life-years in the future less than those at present not because of the timing of these life-years, but simply because a life-year in the distant future implies a long life, which may stand for a satiation effect [44].

No statistically significant interaction effect was found between length of time delay and age group, which suggests that there are no multiplicative effects. However, it is possible that such an interaction exists in other populations, where the events and treatment effects are more closely related to the age.

High belief in treatment necessity in the NCF are known to be associated with adherence and behaviour related to treatment [26]. In this study, greater belief of necessity was associated with increased willingness to initiate therapy; however, adjusting for necessity and concerns did not abolish the effect of an increased DoE effect. The NCF was developed to explore patients’ views on drugs in general and not specifically preventive drugs; consequently the measure is not optimal for use in a study with lay people and a hypothetical scenario. The reason for including the NCF in this study was to capture a general attitude to drugs rather than specific benefits/concerns about the treatment presented in the scenario.

To summarise: the results in this study imply that time magnitudes described as DoE are useful for lay people in decision-making regarding medical treatments. The DoE measure might be an adequate alternative, and/or a complement to other treatment descriptions and a useful tool in shared clinical decision-making. This was the first study of differences in magnitudes for DoE and therefore relatively large differences in the time periods were chosen to explore the concept. Future studies should investigate if patients also are sensitive to minor differences in DoE, and if patient groups with different baseline risks and conditions also are sensitive to minor differences in DoE. This study does not compare variations in DoE magnitudes with variations in other measure such as RRRs or ARRs, therefore no conclusion can be drawn on these matters. It is suggested that future studies address the question on how variations of magnitude of different effect measures relates to each other as well as decision-making in different situations. There is a need to further investigate if time-based measures, such as DoE, may have an advantageous role in clinical decision making for specific patient groups and treatments, especially if they may improve long-term adherence.

### Strengths and limitations

The strengths of this study include the community setting and the randomised approach. This study also has some limitations worth noting. The study used a hypothetical scenario and the study population mainly consisted of lay people (without previous heart disease); thus, the study population were different than typical patient groups. The response rate is reasonable according to that anticipated from mailed questionnaire-based studies, but unfortunately there is no information about non-responders. Further, reporting bias may exist due to the nature of self-reported data.

## Conclusion

Lay people’s willingness to initiate preventive therapy was sensitive to the length of time periods when treatment effect was presented as the time-based measure Delay of Event. The results indicate that Delay of Event is a comprehensible measure of effect, which holds potential value for communicating treatment benefit and shared clinical decision-making.

## Abbreviations

€: Euros
ARR: Absolute risk reductions
BMQ: Beliefs about Medicines Questionnaire
CI: Confidence interval
CVD: Cardiovascular disease
CVM: Contingent valuation method
DoE: Delay of Event
NCF: Necessity-Concern Framework
NNT: Numbers-needed-to-treat OR Odds ratio
PCC: Patient/person-centred care
RRR: Relative risk reductions
SEK: Swedish currency
SPSS: Statistical Package for the Social Sciences
WTP: Willingness to pay
